# Disorder-Specific Profiles of Self-Perceived Emotional Abilities in Schizophrenia and Major Depressive Disorder

**DOI:** 10.3390/brainsci12030356

**Published:** 2022-03-07

**Authors:** Elisabeth M. Weiss, Eberhard A. Deisenhammer, Andreas Fink, Josef Marksteiner, Markus Canazei, Ilona Papousek

**Affiliations:** 1 Department of Psychology, University of Innsbruck, 6020 Innsbruck, Austria; markus.canazei@uibk.ac.at; 2 Department of Psychiatry, Psychotherapy, Psychosomatics and Medical Psychology, Medical University of Innsbruck, 6020 Innsbruck, Austria; eberhard.deisenhammer@i-med.ac.at; 3 Department of Psychology, University of Graz, 8010 Graz, Austria; andreas.fink@uni-graz.ac.at (A.F.); ilona.papousek@uni-graz.ac.at (I.P.); 4 Department of Psychiatry and Psychotherapy A, Hall State Hospital, 6060 Hall in Tirol, Austria; josef.marksteiner@tirol-kliniken.at

**Keywords:** schizophrenia, major depressive disorder, emotion perception, emotion regulation, emotional self-efficacy

## Abstract

Deficits in social cognition are a core feature of neuropsychiatric disorders. The purpose of this study was to compare profiles of self-perceived abilities across the core domains of emotional functioning between patients with schizophrenia (*n* = 22), major depressive disorder (*n* = 31) and healthy participants (*n* = 43) with the Self-report Emotional Ability Scale (SEAS). Profile analyses were used to explore group differences in the overall level of self-perceived effectiveness of emotional functioning and in the patterns in which the four functions of emotion perception and regulation in the intra- and inter-personal domains are arranged to each other. Both patient groups showed significantly lower overall levels of self-perceived emotional functioning compared to healthy controls. Most importantly, we found significant differences between patient groups in their profile patterns. Patients with schizophrenia indicated experiencing difficulties in all investigated domains, but the profile pattern largely matched that of healthy individuals. Instead, the profile of patients with depression was much more accentuated, showing lower perceived effectiveness of emotion perception and regulation in the intra-personal domain compared to inter-personal functions. Our results of disorder-specific emotional deficits may have profound implications for early screening and identification of at-risk populations as well as recovery-oriented interventions.

## 1. Introduction

It is widely acknowledged that proper processing and management of emotional information is important for interpersonal communication, adaptive social behavior, and normal social relationships [1,2,3]. Deficits in social cognition are a core feature of several psychopathologies including schizophrenia and affective disorder [4]. However, until now, only few studies have directly compared deficits in patient groups with different mental disorders. Moreover, the majority of research on affective processing in neuropsychiatric disorders used performance-based measures to capture emotion-related cognitive abilities such as accurately identifying other people’s emotional expressions [5,6,7]. While providing crucial information, these measures picture only part of individuals’ emotional functioning in their daily lives. Beyond the actual capability to process and manage emotions, it needs positive self-appraisals of one’s emotional abilities to put them into action. Emotional self-efficacy is the confidence with which individuals approach the management of emotional events and determines the effective implementation of emotion-related cognitive abilities and knowledge [8,9,10,11,12]. Moreover, research has shown that emotional self-efficacy can be relatively independent from relevant emotion-related cognitive abilities [13,14,15], and is linked to clinical symptoms as well as community functioning in psychiatric patients [16].

While there is general agreement that deficits in the recognition of affect represent an essential part of the social cognitive deficits in patients with schizophrenia and other neuropsychiatric disorders [17,18,19,20,21,22,23], emotional functioning is multidimensional and comprises several components. Accordingly, emotional self-efficacies are specific and refer to particular domains of emotional functioning. Self-appraisals of the core domains of emotional functioning, inter- and intra-personal emotion perception and inter- and intra-personal emotion regulation share a maximum of 20 percent of their variance [24].

Thus, examining and comparing a more complete spectrum of emotion processing may further illuminate differences and similarities across neuropsychiatric disorders.

However, to date there have been only rare attempts to examine profiles of several self-perceived emotional abilities in psychiatric patient groups [25]. There is some preliminary evidence suggesting a link between major depression and deficits in (only) specific domains of emotional self-efficacy [26,27,28]. However, the used scales in this research lack a clear structure and, most important, the key statistical interaction effect of group by domain was not tested, which renders conclusions about specific relationships arbitrary. The latter also applies to two studies comparing patients with schizophrenia [16] and an unspecified psychotic group [29] to patients with affective disorders. Moreover, these studies only evaluated intra-personal emotional competencies. No study so far has specifically compared comprehensive profiles of emotional functioning between patients with schizophrenia and major depression.

For the interpretation of profiles of several emotional abilities, it is vital to use an assessment instrument that allows to assess several domains at the same time and uses the very same assessment method. While this is a virtually impossible task when targeting emotion-related cognitive abilities, this approach is feasible in the case of quantifying emotional self-efficacies, which naturally rely on self-report. In the current study, we used a well-established psychometric instrument for the assessment of emotion perception and emotion regulation in the intra- and the inter-personal domain, which has been successfully employed in several studies of our laboratory [24,30,31,32]. The Self-report Emotional Ability Scale (SEAS) contains only items that focus directly on self-appraisals of emotion-related abilities but not, e.g., on motivational aspects, attitudes, or preferred ways of behaving [14,33,34].

The study objective was to gain a more comprehensive picture of the differences in self-perceived emotional functioning between patients with schizophrenia and major depressive disorder and healthy participants. The analysis of profiles of self-efficacies across the core domains of emotional functioning (inter- and intra-personal emotion perception and inter- and intra-personal emotion regulation) allowed us to explore differences between groups in particular emotional functions as well as whether the diagnostic groups may differ in the patterns in which these functions are arranged to each other. Furthermore, correlations with characteristic psychiatric symptoms of the two disorders (positive and negative symptoms in schizophrenia and depression score in affective disorder) were explored.

## 2. Materials and Methods

### 2.1. Participants

*n* = 112 participants were asked to participate in the study. Eight patients with schizophrenia and four patients with major depressive disorder refused to participate or subsequently decided to withdraw. Four patients with schizophrenia had to be excluded from the final sample because of missing data in the questionnaires.

Finally, fifty-three patients with the ICD-10 diagnosis of schizophrenia (*n* = 22) or major depressive disorder (*n* = 31) as well as 43 healthy individuals were included in the final analysis. All patients were inpatients consecutively recruited from two psychiatric hospitals in Austria. The clinical group was recruited and diagnosed with schizophrenia or major depressive disorder by a licensed psychiatrist according to the ICD-10 [35]. All patients received antipsychotic drugs or antidepressants, respectively. The nonclinical comparison group was recruited by student coworkers at the University of Graz. In the nonclinical comparison group, people with major psychiatric disorders/history of major psychiatric disorders according to the Structured Clinical Interview for DSM-IV-Axis I Disorders (SCID screening) and people who reported having a neurological disease or using psychoactive medication were not included in the study.

The study was conducted in accordance with the 1964 Declaration of Helsinki and was approved by the authorized Ethics Committee (Ethical Code Number: GZ. 39/84/63 ex 2014/15; Approval Date: 1 October 2015). After complete description of the study, all subjects provided written informed consent.

### 2.2. Psychometric Measures

Schizophrenia symptoms were assessed using the Positive and Negative Syndrome Scale (PANSS; [36]). Depressive symptoms were assessed using the Beck Depression Inventory [37].

The SEAS [24,30,31,32,33] includes two subscales for the assessment of self-appraisals of emotion perception and regulation in the intra-personal domain and two subscales for the assessment of emotion perception and regulation in the inter-personal domain, respectively: Perception of one’s own emotions (9 items, e.g., “It often takes a while for me to recognize my true feelings”, “When I am facing a tough task that I absolutely don’t want to fail at, I can easily estimate my fear of it”), Regulation of one’s own emotions (6 items, e.g., “When I am scared of something I can barely do anything about it”, “It’s easy for me to get over a disappointing experience”), Perception of the emotions of others (11 items, e.g., “I can tell immediately if a friend is worrying about something”, “Even in strangers I have no trouble recognizing insincere expressions of emotion”), and Regulation of the emotions of others (11 items, e.g., “I can influence the mood of others very well”, “I have great trouble cheering up an acquaintance who has lost a person close to them”). The items are rated on a six-point Likert scale (1–6). The scale means were used in the statistical analysis, so that the potential range of scores in each scale was 1 to 6. The instrument also allows for the use of two composite scales for the measurement of intra- versus inter-personal functions [33]. Lower scores on the SEAS scales indicate less effective emotion perception or regulation according to participants’ self-appraisal.

### 2.3. Statistical Analysis

Demographic characteristics of the three groups were compared by means of analyses of variance and Pearson’s chi-square tests (see Table 1). Group differences in self-perceived emotion-related abilities were tested by means of profile analysis [38]. For this purpose, a 3 (group; between-subjects factor) × 4 (domain; within-subjects factor) analysis of variance was performed, using the multivariate approach to repeated measures analysis [38]. According to the research questions of the present study, the relevant effects in these analyses are (a) the main effect of group, referring to whether the groups differ in their overall levels of self-perceived effectiveness of emotional functioning; and (b) the interaction effect of group by domain, referring to the question whether group differences may be attributed to specific domains in particular, and the more specific question whether the profiles of the three groups across the four domains are parallel or differ from each other. Bonferroni-corrected *t*-tests were used for relevant post hoc pairwise comparisons. Further analyses tested for correlations between depression severity/positive and negative symptoms and self-perceived intra- and inter-personal abilities (composite scores) in patients with major depression disorder and schizophrenia, respectively (Pearson correlations). Estimates of effect sizes in the profile analysis are reported using partial eta-squared (η_p_^2^), which gives the proportion of variance a factor or interaction explains of the overall variance in the dependent variable. All statistical tests were performed with α = 0.05 (two-tailed).

## 3. Results

Sociodemographic and clinical data are presented in Table 1.

The diagnostic groups did not differ in age (*F*(2, 95) = 0.025, *p* = 0.975) and educational level (χ^2^(4, *n* = 96) = 9.19, *p* = 0.057). However, sex distribution (χ^2^(2, *n* = 96) = 11.98, *p* = 0.003) and depression score (*F*(2, 95) = 58.66, *p* < 0.001) differed between groups.

**Figure 1:**
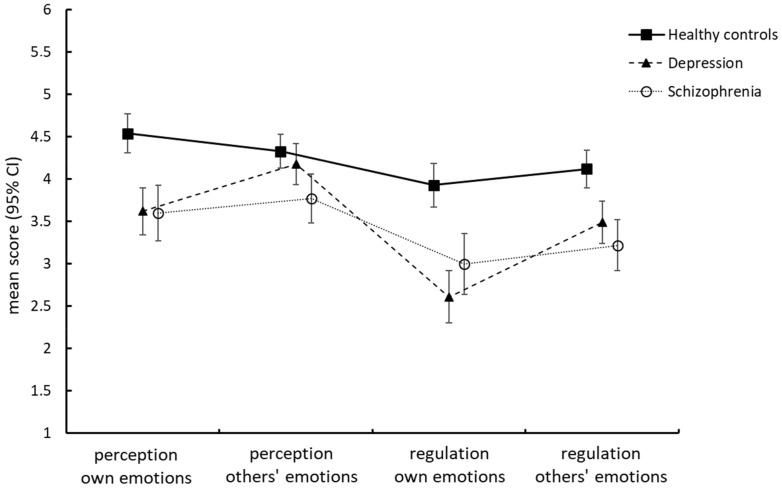
Profiles of self-perceived abilities in four core domains of emotional functioning.

The profile analysis yielded a large main effect of group (*F*(2, 93) = 26.6, *p* < 0.001, η_p_^2^ = 0.36), indicating markedly lower overall levels of self-perceived emotional functioning (averaged across all four domains) in patients with schizophrenia (*M* = 3.40 ± 0.78) and major depressive disorder (*M* = 3.47 ± 0.99) compared to healthy controls (*M* = 4.23 ± 0.75; Bonferroni-corrected post hoc tests, all *p* < 0.001). The significant interaction effect of group by domain (*F*(6, 182) = 4.4, *p* < 0.001, η_p_^2^ = 0.13) additionally indicated non-parallel profiles among the three study groups. Figure 1 shows that the profiles of the two patient groups were not parallel, suggesting that the patterns in which the self-perceived functionalities of the four domains were arranged to each other clearly differed between groups: patients with schizophrenia indicated experiencing difficulties in all four domains, with the pattern in which the domains are arranged to each other very much resembling that of healthy individuals. By contrast, the profile of patients with major depressive disorder was much more accentuated. While the perception of others’ emotions reached the level of healthy individuals (*p* = 1.000), the perceived effectiveness of their efforts to regulate their own emotions was poorest of all groups. The most prominent feature of the depressed patients’ profile was that the functionality of both intra-personal functions was estimated poorer than that of the respective inter-personal functions (i.e., they rated the perception of their own emotions poorer than the perception of others’ emotions, *p* = 0.010, and the regulation of their own emotions poorer than the regulation of others’ emotions, *p* = 0.001). This was not the case in the two other groups (Bonferroni-corrected single comparison tests, all *p* > 0.10). As a result, in patients with major depressive disorder, the discrepancy between the perception of affective information in the social environment and the perceived capacity to regulate one’s emotions was clearly the largest of all groups.

In line with the depressed patients’ profile, in patients with major depressive disorder, higher BDI scores were associated with poorer self-perceived intra-personal abilities (*r* = −0.28, *p* = 0.029), while the correlation with scores in the inter-personal domain was not significant (*r* = −0.05, *p* = 0.676). In patients with schizophrenia, neither the PANSS-positive nor the PANSS-negative subscale showed significant correlations with self-efficacies in the intra-personal (PANSS-positive: *r* = −0.01, *p* = 0.94; PANSS-negative: *r* = 0.18, *p* = 0.24) or the inter-personal domain (PANSS-positive: *r* = −0.17, *p* = 0.28; PANSS-negative: *r* = −0.06, *p* = 0.70).

In addition, the level of education had no influences on any of the domains (*F*(8, 180) = 0.47, *p* = 0.87; multivariate analysis of variance with the four SEAS scales as the dependent variables). Further, we observed no sex differences in any of the SEAS scores (*F*(4, 91) = 0.50, *p =* 0.74; also the four univariate comparisons were not significant; all *p* > 0.10). The latter finding excludes the possibility that the differences in the profiles of the diagnostic groups are only explained by their different gender compositions. The correlation between higher BDI scores and poorer self-estimated intra-personal functions within the group of depressed patients remained unchanged when sex was partialled out (*r* = −0.25, *p* = 0.05).

## 4. Discussion

In the current study, overall levels of self-perceived emotional functioning were higher in the healthy control group than in patients with schizophrenia and patients with major depressive disorder. Even more important, we found differences between the two patient groups in the profiles of their self-perceived emotional functioning. Patients with schizophrenia indicated experiencing difficulties in all four domains, but the overall pattern of findings closely matched that of healthy individuals. Furthermore, we found no correlations between the severity of clinical symptoms measured with the positive and the negative PANSS subscales and the perceived effectiveness of emotion perception and regulation processes in schizophrenic patients. The finding of overall poorer self-perceived emotional functioning in schizophrenia patients replicates prior findings of impairments in self-estimated perception and regulation of one’s own emotions [16] and related concepts such as alexithymia [39,40] or dispositional mindfulness [41]. Overall, the pattern of findings also matches emotion processing impairments displayed on measures of emotion-related cognitive abilities such as facial affect recognition (for a review see [5]) and in neurophysiological emotion regulation tasks [20,42,43].

By contrast, we found that the profile of patients with depression was much more accentuated, showing markedly lower self-perceived effectiveness in the intra-personal domain compared to inter-personal abilities. Especially, the perceived capacity to regulate one’s emotions was low in this group. In line with the depressed patients’ profile, in patients with major depressive disorder, higher BDI scores were only associated with poorer self-efficacy in the intra-personal domain, but not in the inter-personal domain. These findings correspond to previous studies suggesting that the dimension of emotional regulation is the core feature in the association between self-perceived emotional abilities and depression, and that better emotional regulation is related to elevated psychological well-being, happiness, lower perception of stress and a better quality of life [44,45]. Interestingly, depressed patients in our study reached the level of healthy individuals in the subscale “perception of others’ emotions”. Meta-analyses suggested that there is a significant theory of mind (ToM) deficit with medium effect size in major depressive disorder [46], but there only exists a small impact of depression on facial emotion recognition capacity [47]. However, it is important to note that inter-personal emotion perception in the instrument used in the present study refers to the self-rated susceptibility to affective information in the social environment, that is, to the degree to which a person tends to perceive the emotions of other people [30,48]. It does not explicitly refer to primarily cognitive processes subsumed under the terms of theory of mind or cognitive perspective-taking, or to emotion recognition in terms of the correct identification of emotions.

Our results, showing a combination of high tendency to perceive affective information and poor emotion regulation in depressed patients, nicely resemble previous findings by Ciarrochi et al. [49], who showed that emotionally perceptive people appear to be more strongly impacted by stress, leading to higher levels of depression, hopelessness and suicidal ideation than their less perceptive counterparts. Paired with poor emotion regulation capacity, this feature may make individuals particularly vulnerable.

Our study has several limitations. First, the sample size is small, especially for the patients with schizophrenia, and at the time of the investigation all patients had an inpatient status and received antidepressant and/or antipsychotic drug therapy. We therefore cannot exclude possible effects of psychopharmacologic drug intake on self-perceived emotional functioning/emotional self-efficacies. Second, both schizophrenia and depression are syndromes characterized by a great heterogeneity, which is expressed in terms of their symptomatic manifestations, course of illness, regularity of phases, comorbidities, and also duration of illness. In this study, we did not control for all these factors and, therefore, can only make cautious conclusions about the influence of clinical characteristics of patients with schizophrenia or depression on their emotional self-efficacies. Third, in the current study we did not investigate a potential influence of non-social cognition on self-perceived emotional functioning/emotional self-efficacies.

## 5. Conclusions

Taken together, our results showed that patients with schizophrenia feel impaired in all aspects of emotional functioning, whereas patients with major depressive disorder showed a more distinct pattern of findings. The most prominent feature of the depressed patients’ profile was that the functionality of the intra-personal abilities of emotion perception and regulation was perceived poorer than that of the respective inter-personal functions. Therefore, in order to achieve a more comprehensive understanding of the social cognitive deficits in different psychiatric disorders, the current study encourages to include not only measures of emotional self-efficacy, but also to include a broader assessment of emotion processes that go beyond single concepts typically used in social cognitive studies such as accurate perception of the emotions of others, the ability to recognize important social cues or theory of mind. By assessing all four cross-combinations of the core domains of emotional functioning at the same time with one and the same assessment method, we were able to comprehensively compare the profiles of self-perceived emotional functioning and explore differences in the patterns in which these functions are arranged to each other. Our results of disorder-specific patterns of emotional deficits might have profound implications for early screening and identification of at-risk populations as well as recovery oriented interventions. Deficits in social cognition are considered to be core features of schizophrenia and major depressive disorder. Hence, they are not only apparent during acute episodes of the disorders and endure when patients are in remission, but might additionally represent a risk factor for the transition into psychiatric illness in ultra-high-risk people. Furthermore, knowledge on disorder-specific deficits in emotional self-efficacies can be used to improve treatment programs and to develop more precise and effective psychotherapeutic programs which integrate interventions designed to improve disorder-specific impairments in emotional competency.

## Figures and Tables

**Table 1:** Sociodemographic and clinical data.

	Participant Groups		
	C (*n* = 43)	S (*n* = 22)	D (*n* = 31)
Age (years)	39.5 (13.4)	39.1 (15.29)	39.9 (11.2)
Sex (Male/Female	23/20	16/6	8/23
* Education			
1: Less than high school	62%	59%	74%
2: High school graduate	26%	41%	10%
3: Some college	12%	0%	16%
BDI	3.7 (3.8)	17.6 (12.2)	26.7 (11.7)
PANSS			
PANSS-Positive scale	-	20.5 (9.9)	-
PANSS-Negative scale	-	20.9 (10.4)	-
PANSS General scale	-	41.8 (20.0)	-

M = mean; SD = standard deviation; C = healthy controls; S = patients with schizophrenia; D = patients with major depression; BDI = Beck Depression Inventory PANSS = Positive and Negative Syndrome Scale; * Level of education was measured in terms of highest level of education completed.

## Data Availability

The datasets generated during and/or analyzed during the current study are available from the corresponding author on reasonable request.
